# Chicago Medical Society; Meeting August 16

**Published:** 1886-10

**Authors:** 


### SOCIETY REPORTS.

---

CHICAGO MEDICAL SOCIETY.

*Regular Meeting, August 16, 1886.*—E. J. DOERING, M.D., PRESIDENT, in the chair.

PROFESSOR F. E. WAXHAM presented a membranous cast of the trachea and larynx. The specimen presented was removed from a child nine years old. This cast had remained in the larynx and trachea for several months. The patient was brought to him from Minnesota by Dr. McDavitt, of Winona. The history is as follows: The child claims that in April she swallowed a hedge thorn whilst away from home. She was at once taken with suffocation, and twenty-four hours afterwards was operated upon when at the point of death, by Dr. McDavitt, who performed tracheotomy. The child was unable to breathe through the natural passages after the introduction of the tracheotomy-tube, although many attempts were made to remove it. It seemed impossible for the child to get a breath through the natural passages when she was brought to Professor Waxham. Upon laryngoscopic examination the larynx seemed to be closed, but digital examination revealed a very small opening. Into this opening a small sound was passed, and this was followed by one of the smallest sized intubation-tubes, this by a larger one, and finally the largest sized tube was introduced. It could not be passed on account of the tracheotomy-tube, and violent vomiting ensued, when the tube and this cast were ejected. After the ejection of this membrane a large sized tube was introduced and pressed down into position, as the tracheotomy tube was removed. This gave the child great comfort. She remained comfortable after the introduction of the tube, took several glasses of milk during the afternoon, and in the evening was taken to the train and returned to Minnesota, the intubation-tube remaining in the larynx to be removed by Dr. McDavitt in the course of a few days.

PROFESSOR JOHN B. HAMILTON, of Washington, D. C., read a paper on

THE RADICAL CURE OF INGUINAL HERNIA, in which he said that the ablest surgeons from the earliest times to our day have given much attention to this subject. Dr. Baxter's tables show that out of 334,321 recruits and substitutes examined by the recruiting officers during the war of the rebellion, more than 17,000 were rejected on account of hernia. The London Truss-Society, during the first twenty-eight years of its existence, issued over 83,000 trusses. Two factories in Philadelphia manufacture and sell from 216,000 to 250,000 per annum.

Celsus was the first surgeon to have definite ideas about the operation for the cure of hernia; he used cauterization and a bandage. Ligature of the sac has been practiced from an early day. Maupas performed gastrorraphy. Lanfranc, in 1296, favored castration. Ambrose Pare was the first to absolutely abandon castration; he used astringents. Freytag was the first to practice dilatation of the rings in strangulated hernia. Nicholas le Quin, of Paris, introduced the truss about the year 1660, but it was not until the middle of the eighteenth century that surgeons began to cut off the gangrenous portion of intestine in cases of strangulated hernia. The galvano-cautery was proposed by Dr. John C. Minor, of New York. Galaud, in 1878, favored the elastic ligature. The practice of scarification is a very ancient one, and has been brought down to recent date with excellent results. Guerin was probably the first to practice subcutaneous scarification. Invagination has for its object the occlusion of the inguinal ring by the fascia, and sometimes by the integuments. The late Dr. George Allen, of Springfield, Illinois, reported fifty cases cured by Gerdy's method. Wood's method not only invaginates the fascia, but draws together the pillars. All of these methods have their greatest successes in small hernias and bubonoceles, and are absolutely valueless in those which are so large that ordinary invagination will not occlude the opening. Dr. Alexander stated, in 1883, that he had performed the radical cure thirty times without any deaths. Wood reports 339 cases without special antiseptic precautions; 96 were cured, 7 died, and 59 failed; in the remainder the result could not be ascertained. Accidents may follow Wood's operation as well as others, such as sloughing, peritonitis, and tetanus. From official reports on file in the Marine Hospital Bureau at Washington, it appears that in Calcutta, India, in 1884, out of a total number of deaths from all causes of 1,293, nearly nine per cent. were due to tetanus. Professor Hamilton favored an operation in all cases affording a reasonable prospect of cure, and thought all cases of bubonocele should be operated upon.

PROFESSOR D. W. GRAHAM said he felt he expressed the sentiments of every member present when he said that it is probably not possible to find in the English language as complete and satisfactory a review of the history of the various operations for the radical cure of hernia as Dr. Hamilton has given us in this paper. Certainly it has been gratifying and instructive to us all to listen to it. Some of the figures which Dr. Hamilton quotes give us an idea of the great prevalence of hernia. He believed reliable statistics show that on an average about every fifteenth or sixteenth individual in civilized communities suffers from some form of it. When we remember this great prevalence, and when we remember that every subject of a hernia sustains thereby a certain amount of disability, and that very many are entirely disabled by it, the subject of the radical cure of hernia assumes an importance not always accorded to it. He thought the author of the paper took a rather highly colored view of the future of these operations. It is not probable that this generation, at least, will be able to use the expressions he thinks surgeons will be using some day in Chicago. However, this degree of success is to be looked forward to and attained if possible. In regard to the modern methods of operating, he understood the distinctive features of Wood's method to be that it is almost entirely subcutaneous, that he uses wire, and that he allows the sac to remain in the canal as a kind of plug. So far as he knew, this method is not practiced in this part of the country to any great extent. In Mr. Wood's hands it seems to be successful in permanently curing a considerable majority of those operated upon, and in decidedly benefiting a good many who are not permanently cured. His statistics show that there is almost no danger to life from the operation itself, at least in his hands. Any method which will give such results, and at the same time involves so little risk to life, must be a good operation.

The open method, the one chiefly practiced in this country, according to his observation, contemplates strict attention to antiseptic details and the avoidance of suppuration. It involves a little more risk to life from the operation *per se*, but to compensate for this it would seem to give, theoretically at least, a larger percentage of permanent and complete cures. The modifications or varieties consist chiefly in dealing with the sac. Although MacCormac claims that unless the rings are wide there is no advantage in attempting to close them, yet it seems that the best way to treat the sac is to excise a section of the neck between two ligatures, pushing the stump into the abdominal cavity and allowing the body of the sac to remain, unless it is small and loosely adherent, when it may be extirpated. After any of these operations there remains the funnel-like depression on the inner surface of the abdominal wall, which favors a recurrence of the hernia, however efficiently we may have obliterated the sac and closed the rings. MacEwen, of Glasgow, in a recent article describes and advocates a plan he has devised for obliterating the depression. He utilizes the sac by dissecting it from its surrounding attachments, putting a suture through it from side to side beginning at the lower end, thus making a corrugated pad, which he pushes through the internal ring into the cavity, after first separating the peritoneum for a little distance around the ring with the finger. This makes a convexity on the inner surface of the abdominal wall, when there would otherwise be a concavity. This modification appears to be a real improvement, but the practical value of it is as yet largely conjectural. By way of adding something from an historical standpoint, there might be mentioned what was not alluded to in the paper, that electrolysis has been used and advised to set up a plastic inflammation in the inguinal tract. He believed this suggestion came from a Cleveland surgeon, whose name he had forgotten.

This is the same in principle, of course, as the use of subcutaneous injections, and he should think would be preferable to the injections, if he were to judge of it without having tried it.

PROFESSOR MOSES GUNN said: This subject is of intense interest, and we are very much indebted to Dr. Hamilton for the exhaustive review he has given us. We have to consider what are the best methods for operating with the intent of effecting a radical cure. He was inclined to discard all the old invaginating processes. In the first place, the invaginated portion is always liable to subsequent prolapse; in the next place, it is always a foul mess. It won't do to simply remove the cuticle; all the organs of the skin must be destroyed. The skin contains the hair, sweat and sebaceous follicles, and unless they are destroyed they will continue their work, and thus accumulate material for decomposition. In order to make a success you are obliged to do more than destroy the cuticle. You will have to destroy the skin, and that is a very slow process and exceedingly nasty. Therefore he was inclined to repudiate all invaginating processes. He repudiates also all of the subcutaneous processes, for they are blind procedures, and he would not adopt them where an open operation could be as well and even more advantageously and safely resorted to. He believed the best and surest method of trying to effect a radical cure of hernia is the open method. This method should be performed in every case where an operation is made for the relief of a strangulated inguinal hernia. Just as soon as the operator has opened the neck of the sac and restored the prolapsed viscera or viscus, he should close up the wound. He should begin at the topmost portion of the sac, and with a curved needle and catgut take it up and ligate it as near to the internal ring as he can. Then he should drop down about half an inch lower and ligate again, and so on down through the canal. He should then approximate the pillars at and above the external ring as closely as possible and close the outside tissues. Thus the operation which is made for the relief of the accident should be made an operation for radical cure. It can be done more effectually at that time than at any other.

Again, the physician is called upon to make an operation for a radical cure; the patient comes to him complaining of the truss, which has become ineffectual, the hernia escaping in spite of it, and his life becoming a misery to him, and he asks if something cannot be done to make a radical cure. The answer is, "yes"; but the question is, by what method shall we make it? Choose the open operation, practiced with all antiseptic precautions. Cut down upon the parts, separate and dissect out as well as you can the neck of the sac, the hernia having been reduced; ligate the sac and cut out a portion. Thrust the stump back into the canal and approximate the pillars, closing them tightly and keeping the external ring tightly closed. So much for the method; now for the prospect of success. How much right have we to expect what might justly be called a radical cure? By the term radical, he meant permanent. In what proportion of cases can we expect to have a permanent cure, so that the patient will never have hernia again? What is hernia, and who have it? He thought he could safely say that the typical man never has hernia. When the true type in development has been attained and the abdominal walls closely woven together, they are proof against such accidents and there will be no hernia. Hernia is the result of the imperfect development of the abdominal muscles and aponeuroses. When that imperfect anatomical development obtains in the patient, the abdominal muscles are thin and flabby, and in such cases we get hernia.

Nor can we in such a case by an operation make a man better than his Creator made him, but if we can make him as good as he was we may congratulate ourselves. After we have operated and closed up this weak point as well as possible, the very best result that we can expect is that we have made the man as good as he was before he had hernia; but, if he was weak enough to have hernia from certain exciting causes, he will be weak enough to have it under similar circumstances again. If his hernia is brought on by lifting and straining, the same exciting cause will bring it on again, and under these circumstances a radical cure is only measurably radical. We should tell the patient after operation that if he will be more careful and take no violent exercise, he may hope for exemption from hernia.

In other cases, the patient has an old and immense hernia and cannot wear a truss; we operate upon him and can say to him that if he will be more careful and wear a truss, he can be tolerably comfortable for the rest of his life. Such, he apprehended, is the true aim and scope of operations for the radical cure of hernia; such are the precautions that should be given patients, as they must become our coöperators in order to make this operation a success; and with such coöperation and conscientious efforts on our own part, radical cure of hernia becomes a standard and important operation, as important as the subject itself, which, as we have seen, is of immense importance on account of the great dissemination of the disease.

DOCTOR E. F. WELLS said: Dr. Hamilton has certainly read a very interesting and extensive paper. There is one point in particular mentioned by the author, namely, that he advocates an operation in all suitable cases where an operation is not distinctly indicated. Every practitioner of large experience must certainly have met with many cases in which the truss had been applied resulting in a cure, and he thought the truss should not be stricken entirely from the methods of obtaining a radical cure of hernia.

PROFESSOR HAMILTON in closing the discussion said he need not say that he was extremely gratified to find such substantial unanimity of sentiment as to the propriety of operation—nay, as to the necessity of operation, but there can be no doubt as to the necessity of further statistics on the subject. In regard to invagination, he thought a reading of the paper would not show that he advocated the method of invagination recommended by Gerdy. In the recent open method there is no invagination. Under the original Wood's method, the subcutaneous fascia only was pushed up under the ring; by the open method we cut down directly on the sac. This open method is really a combination method, because it brings together the pillars and takes care of the sac. Statistics are necessarily unreliable as to the ultimate permanency of the cure of these cases. The best statistics are those shown by the Swedish Hospital, where out of 300 cases a large percentage of recoveries is shown, and if statistics are worth anything in determining the success of a method, we must place some reliance on these. It would be well to have patients come back every year for the purpose of re-examination.

Professor Fenger, if he correctly understood him, speaks of the influence of suppuration in curing these wounds by letting them heal from the bottom, but in the various subcutaneous operations that is exactly what it is intended to avoid. There is no doubt that suppuration will make a radical cure of hernia if the patient's strength lasts, and the suppuration does not extend into the abdominal fascia. That was the method by which the old red-hot irons accomplished their purpose. The mineral acids produced a radical cure by the destruction of the tissue and healing from the bottom. The seton also performed a cure, but it has so many disadvantages that it is not to be compared with those procedures that stop the inflammatory processes short of the decomposition or death of the exudate. In regard to operating on children, he thought the argument cannot be regarded as sound that we should not operate on them on account of the difficulty of keeping a bandage on, for surely if any cases are to be benefited by an operation for radical cure, they are those in which the patient is young enough to grow—in which the tissues can be brought together and retained with great hope of a permanent cure. Everybody knows that cases do recover by the use of the truss, but the proportion, he believed, is less than by any other method. As stated by Professor Gunn, it is found that a majority of operations for strangulated hernia are, in effect, really operations for the radical cure, and there are more than five cures from operations to one after application of the truss. And when we remember that there are 250,000 trusses manufactured per annum in Philadelphia alone, he doubts very much if it can be shown that trusses have even a fair percentage of recoveries following their use.

PROFESSOR WM. T. BELFIELD reported a case of

SUPRA-PUBIC CYSTOTOMY WITH EXTRACTION OF LARGE CALCULI, AND CORROSIVE SUBLIMATE POISONING.

The patient was a feeble, emaciated man, 71 years old, who for nine years had suffered from cystitis of steadily increasing severity, caused, as was supposed by his various physicians, by prostatic enlargement. He had been sounded for stone a year ago under chloroform, but with negative result. For two years he had been unable to empty the bladder except by catheter. He refused permission to introduce the sound because convinced that he had no stone, but was anxious to have an operation for the removal of the prostatic enlargement.

June 7, supra-pubic cystotomy was undertaken for the purpose of removing by galvano-cautery that portion of the prostate which was assumed to project into the bladder. The introduction of the sound under ether revealed a large stone; the usual incision was made; the finger in the bladder found two calculi, one behind the prostate, the other adhering to the fundus of the bladder, each about as large as a walnut. The first stone was crushed and the second with much difficulty removed entire. The patient was so collapsed that no attempt was made to remove the prostatic outgrowth, which could have been accomplished without much difficulty, and the following day the temperature was 100.5° F., the highest observed. On the third day it was normal and so remained. The wound was irrigated once daily with a bichloride of mercury solution. The progress was entirely favorable until the eleventh day, when there began a severe diarrhœa with much rectal pain and tenesmus, and later the evacuations were tinged with blood and the patient complained of a metallic taste. Sublimate poisoning was recognized and the solution discontinued. Temporary improvement followed, but death ultimately resulted on the thirty-sixth day after operation. No autopsy was permitted, but a hasty examination of the abdominal contents was made. Peritoneum, kidneys and bladder were normal, except that the latter was much hypertrophied. The intestines could not be opened; the calculi weighed 2 ounces and 6 drachms.

THE DEATH OF PROFESSOR F. H. HAMILTON.

PROFESSOR TRUMAN W. MILLER offered the following resolution, which was adopted:

"WHEREAS, This Society has learned with deep regret of the death of Dr. Frank Hastings Hamilton, of New York, and

"WHEREAS, In his death, the United States has lost one of its most distinguished surgeons; one of its ablest teachers; one of the purest patriots, and, in his private life, one of the most amiable of men; therefore, be it

"*Resolved* by this Society, That we hereby give public testimony to the many virtues of the deceased, and that we tender to his family the assurance of the profound sympathy of this Society with them in the hour of their affliction; that a copy of these resolutions be furnished them, and that a copy be spread upon the records."

---

CHICAGO MEDICAL SOCIETY.

